# Greater Total Antioxidant Capacity from Diet and Supplements Is Associated with a Less Atherogenic Blood Profile in U.S. Adults

**DOI:** 10.3390/nu8010015

**Published:** 2016-01-04

**Authors:** Kijoon Kim, Terrence M. Vance, Ock K. Chun

**Affiliations:** Department of Nutritional Sciences, University of Connecticut, Storrs, CT 06269, USA; drkijoon@gmail.com (K.K.); terrence.vance@uconn.edu (T.M.V.)

**Keywords:** total antioxidant capacity, supplements, blood lipids, NHANES, cardiovascular disease

## Abstract

Evidence from epidemiologic studies has shown that total antioxidant capacity (TAC) in the diet might be inversely associated with stroke, heart failure, and inflammatory biomarkers. However, studies on the association of TAC from both diet and supplements with cardiovascular disease (CVD) risk factors in the U.S. population are lacking. This cross-sectional population-based study aimed to investigate the association of TAC with both diet and supplements with CVD risk factors among 4039 U.S. adults in National Health and Nutrition Examination Survey (NHANES) 2007–2012. TAC from both food sources and dietary supplements was estimated from two 24-h dietary recalls using the NHANES supplement ingredient database, United States Department of Agriculture (USDA) proanthocyanidin, flavonoid, and isoflavone databases. Top contributors to TAC were tea, antioxidant supplements, vegetable mixture, orange juice, berries, and wine. Antioxidant supplement users had 1.6 times higher TAC than non-users. Greater TAC was associated with reduced triglycerides (TG) (−1.39% change; 95% CI = −2.56 to −0.21), TG to high-density lipoprotein cholesterol (HDL-C) ratio (−2.03% change; 95% CI = −3.45 to −0.60), HDL-C (0.65% change; 95% CI = 0.07 to 1.23), insulin (−1.37% change; 95% CI = −2.64 to −0.09), homeostasis model assessment of insulin resistance (HOMA-IR) (−1.57% change; 95% CI = −3.02 to −0.09) and C-reactive protein (CRP) (−0.83% change; 95% CI = −1.29 to −0.38) after adjusting for potential confounders. There was no significant association between TAC and waist circumference, BMI, blood pressure, low-density lipoprotein cholesterol (LDL-C), total cholesterol (TC), and fasting glucose. The findings of this study support the hypothesis that an antioxidant-rich diet and intake of supplements are beneficial to reduce CVD risk.

## 1. Introduction

Oxidative stress and inflammation have been implicated in cardiovascular disease (CVD) etiology. Reactive oxygen species (ROS) may play a key role in atherogenesis by oxidizing low-density lipoproteins [1]. Greater consumption of fruits and vegetables has been shown to be associated with a reduced risk of CVD [2], and the beneficial effects of these foods might be attributable to the antioxidant properties of phytochemicals and vitamins rich in fruits and vegetables [3,4,5]. However, this is not consistent with the findings of randomized controlled trials on antioxidant supplements [6,7]. The reasons for the difference are not clear, but one possibility is that such observational and intervention studies might fail to measure antioxidant intake accurately due to measurement error or the combined additive and/or synergistic actions of all antioxidants present in the diet consumed [8,9]. Given that single antioxidants may not reflect total antioxidant power in the diet, dietary total antioxidant capacity (TAC) is a cumulative measure of antioxidants present in the diet, and can be considered an index of antioxidant intake [10]. TAC has also been shown to be an indicator of diet quality [11].

Several research groups have reported an inverse association between TAC and risk of stroke and heart failure [12,13,14]. A recent population-based prospective cohort study with the Swedish Mammography Cohort showed that TAC is inversely associated with total stroke among CVD-free women and hemorrhagic stroke among women with CVD history [12]. In an Italian cohort of the European Prospective Investigation into Cancer and Nutrition (EPIC) study using TAC values of 150 food items measured by Trolox equivalent antioxidant capacity (TEAC) assay, TAC was inversely associated with ischemic stroke cases [13]. Two Italian crossover intervention studies reported the beneficial effects of a high-TAC diet on reduction of systematic inflammation and improvement of endothelial function [15,16]. Hermsdorff *et al.* [17] reported an inverse association between TAC and glycemia, total cholesterol to high-density lipoprotein cholesterol ratio (TC/HDL-C ratio), triglycerides (TG), oxidized low-density lipoprotein cholesterol (LDL-C), and waist circumference in 266 healthy young subjects in Brazil and Spain. In these studies, TAC was estimated based on published TAC databases with a limited number of food items. TAC values in published TAC databases were determined by an experimental method, in which commonly consumed and selected food items were measured by the TAC assays. The limitation of this approach is that the low coverage of the database results in underestimation of TAC, since TAC from foods that are not listed in the database is recorded as zero. Furthermore, considering the high contribution of supplements to TAC and the prevalence of antioxidant supplement use in the United States [18], dietary supplements should be included in the estimation of TAC. Our research group has documented the baseline dietary TAC of U.S. adults from NHANES 2001–2002 by combining the USDA flavonoid, proanthocyanidins, and supplement databases [19]. We found that TAC from both diet and supplements was associated with decreased serum C-reactive protein (CRP) and plasma homocysteine [20,21], implying potential applicability of TAC in predicting CVD risk.

However, studies on the association of TAC from both diet and supplements with blood risk factors for CVD are limited [17]. Therefore, the present study aimed to investigate the association of TAC from both diet and supplements with blood CVD risk factors among U.S. adults by utilizing NHANES 2007–2012 and USDA flavonoid databases. We hypothesized that greater dietary TAC is associated with more favorable blood CVD risk factors.

## 2. Methods

### 2.1. Study Population

A cross-sectional study was conducted with 4039 U.S. adults aged 19 years or older from the NHANES 2007–2012 [22,23,24]. We excluded pregnant or breastfeeding women (*n* = 112), those with dietary recalls coded as unreliable or incomplete (*n* = 1086), those whose dietary recalls were coded as “much more than usual” or “much less than usual,” or those who answered yes to “Are you currently on any kind of diet, either to lose weight or for some other health-related reason?” (*n* = 2256) and those who had an extreme TAC value greater than 10,000 (*n* = 3).

### 2.2. Estimation of Antioxidant Intake from Diet and Supplements

This study used the USDA Database for the Flavonoid Content of Selected Foods, version 3.1 [25], the USDA Database for the Isoflavone Content of Selected Foods, version 2.0 [26], and the USDA Database for the Proanthocyanidin (PA) Content of Selected Foods [27]. We used the USDA PA database, including values for 205 food items for monomers, dimers, and trimers. The combined flavonoid database consisted of 32 dietary flavonoid compounds classified by flavonoid subclasses: flavonols, flavones, flavanones, flavan-3-ols, anthocyanidins, isoflavones, and proanthocyanidins. This combined database was expanded to include additional foods as described in a previous publication [28]. Dietary flavonoid intake was estimated by combining the flavonoid, isoflavone, and proanthocyanidin databases with the food consumption data of the NHANES 2007–2012. Average daily nutrient intakes were estimated from two days of 24-h dietary recall data in the NHANES 2007–2012.

Dietary supplement data on the usage of vitamins, minerals, herbal and other dietary supplements, as well as non-prescription antacids, were collected from 24-h dietary recall interview from NHANES 2007–2012. In order to calculate the TAC from supplements, a nutrient composition table of supplements was made using the ingredient information dataset of the NHANES dietary supplement database. Average daily TAC from supplements was estimated by combining the nutrient composition table with two days of supplement consumption data. The present study includes vitamin C, vitamin E (α-tocopherol, β-tocopherol, γ-tocopherol, δ-tocopherol), carotenoids (α-carotene, β-carotene, cryptoxanthin, lutein, lycopene, zeaxanthin), and flavonoids (flavonols, flavones, flavanones, flavan-3-ols, anthocyanidins, isoflavones) to estimate TAC from supplements. In this study, “antioxidant supplement non-users” were defined as those who had zero contribution to TAC from supplements, while “users” were defined as those who had any positive TAC contribution from supplements.

### 2.3. Estimation of Total Antioxidant Capacity from Diet and Supplements

The antioxidant capacity of individual antioxidants was expressed as vitamin C equivalents (VCE) using the 2,2′-azino-*bis*(3-ethylbenzothiazoline-6-sulphonic acid) (ABTS) assay [29]. These antioxidant nutrients include 29 flavonoids (cyanidin, delphinidin, malvidin, pelargonidin, peonidin, petunidin, (+)-catechin, (+)-gallocatechin, (−)-epicatechin, (−)-epigallocatechin, (−)-epicatechin 3-gallate, (−)-epigallocatechin 3-gallate, theaflavin, theaflavin 3-gallate, theaflavin 3′-gallate, theaflavin 3,3′-digallate, thearubigins, eriodictyol, hesperetin, naringenin, apigenin, luteolin, isorhamnetin, kaempferol, myricetin, quercetin, daidzein, genistein, and glycitein), three PA (monomers, dimers, and trimers), six carotenoids (α-carotene, β-carotene, β-cryptoxanthin, lutein, lycopene, and zeaxanthin), four vitamin E (α-tocopherol, β-tocopherol, γ-tocopherol, and δ-tocopherol) and vitamin C. As thearubigins have been reported to be potent antioxidants like theaflavins [30], thearubigins were assigned the same antioxidant power as theaflavins. The individual antioxidant intake of subjects was estimated by multiplying the contents of individual antioxidants by the daily consumption of each food and supplement. Individual antioxidant capacity was calculated by multiplying the intake of each antioxidant compound by its respective antioxidant power. Theoretical TAC was determined by summing the individual antioxidant capacities. To determine the top sources contributing to TAC, individual foods and food groups were extracted or classified based on the Food Surveys Research Group (FSRG)-defined food coding scheme for use in reporting estimates of food intake in past USDA food surveys [31]. Vegetable mixture was defined as raw and cooked vegetables and their mixtures other than white potatoes, dark green and deep yellow vegetables, tomatoes, lettuce, green beans, corn, peas, and lima beans.

### 2.4. CVD Risk Factors

Waist circumference, height, weight, and blood pressure were collected in the mobile examination center [32]. Body mass index (BMI) was calculated from measured height and weight values (kg/m^2^). Serum TC, TG, HDL-C, and CRP were measured as described in the NHANES Laboratory Procedures Manual [33]. LDL-C was calculated by the equation: LDL = TC − HDL − 0.2 × TG [33]. Homeostasis model assessment of insulin resistance (HOMA-IR) is an index to assess insulin resistance and was calculated as (fasting serum glucose mg/dL × Insulin μU/mL)/405 [34]. The ratios of TC to HDL-C and TG to HDL-C were included as risk factors, as elevations in either of these two ratios may correspond to greater CVD risk [35,36].

### 2.5. Statistical Analysis

All statistical analyses were performed using SAS software, version 9.4 (SAS Institute Inc., Cary, NC, U.S.), using SAS survey procedures and the appropriate weight, strata, domain, and cluster variables to account for the complex survey design. TAC was log transformed and adjusted for average energy intake by the residual method [37].

The frequencies according to energy-adjusted quartiles of TAC were estimated across sociodemographic and lifestyle factors. Participants were grouped into quartiles based on TAC and the means of CVD risk factors were calculated across quartiles of TAC. Participants were classified by poverty income ratio (PIR) of less than or equal to 1.3 and more than 1.3. Based on the number of any kind of alcoholic beverage per day, alcohol consumption was defined with no consumption of drink as none, no more than 2 drinks/day for men and no more than 1 drink/day for women as moderate, and more than 2 drinks for men and more than 1 drink for women as high intake [38]. Positive smoking status was defined as having at least 100 cigarettes per year with current smokers defined as those who had not quit (heavy ≥15 cigarettes per day and light <15 cigarettes per day) and former smokers as those who had reported quitting by the time of interview.

Physical activity was based on weekly minutes of walking/bicycling, moderate/vigorous recreational activities by multiplying the number of days per week by the average minutes of activities on a typical day [39]. MET-minutes per week were calculated by multiplying weekly minutes of activities by the assigned metabolic equivalence of tasks (METs) values. Subjects who reported no walking, no bicycling, or no other moderate/vigorous recreational activities for at least 10 min continuously in a typical week were defined as inactive. The use of blood pressure medication was defined as taking prescribed medicine for high blood pressure or cholesterol. The use of diabetes medication was defined as taking insulin or diabetic pills to lower blood sugar. Intake of major foods according to quartile of TAC was estimated.

In regression models, all CVD risk factors were log transformed after inspecting residual plots. As the model was fit with both the predictor and outcome on the logarithmic scale, the slope from the regression model was used to calculate the percent change in CVD risk factor for a 100% increase in TAC. Multivariate model 1 was adjusted for the following variables: total energy intake, age, gender, ethnicity, physical activity, PIR, alcohol consumption, smoking status, blood pressure medication, diabetes medication, and BMI. Multivariate Model 2 included all variables of Model 1, and additionally adjusted for intake of saturated fatty acids and fiber. The multivariate model of BMI was adjusted for all variables except BMI and all quartiles of TAC were energy adjusted. As CRP data from NHANES are currently available only until 2010, this study included CRP data of NHANES 2007–2010 (*n* = 2794). All *p*-values reported are two sided (α = 0.05). No multicollinearity was detected with variance inflation factors less than 2.0 for all variables in fully adjusted models.

## 3. Results

Table 1 shows sociodemographic and lifestyle characteristics of study participants by energy-adjusted quartile of TAC. The subjects in the highest quartile of TAC were more likely to be women, older, white, antioxidant supplement users and had high levels of income and a low BMI. The subjects in the lowest quartile of TAC were more likely to be younger, current smokers, physically inactive, antioxidant supplement non-users, and had low levels of income, lower fiber intake, higher intake of saturated fatty acid, and a higher BMI.

**Table 1:** Sociodemographic and lifestyle characteristics by energy-adjusted quartiles of total antioxidant capacity among U.S. adults in the NHANES 2007–2012 (*n* = 4039).

Characteristics	*N*	Total Antioxidant Capacity (mg VCE/day) (Range, Median)			
		Q1 (*n* = 975)	Q2 (*n* = 1001)	Q3 (*n* = 1035)	Q4 (*n* = 1028)
		(0.5–216.6, 74.1)	(65.6–466.8, 188.9)	(142.3–1199.8, 428.9)	(379.7–9362.9, 1219.4)
		*n* (%)	*n* (%)	*n* (%)	*n* (%)
Gender					
Men	2071	545 (55.2)	504 (52.7)	520 (50.9)	502 (45.7)
Women	1968	430 (44.8)	497 (47.3)	515 (49.1)	526 (54.3)
Age (year)					
19–30	775	251 (27.5)	208 (23.5)	179 (16.8)	137 (14.7)
31–50	1333	365 (42.6)	330 (37.2)	335 (37.6)	303 (34.1)
51–70	1267	261 (23.6)	305 (28.1)	327 (32.5)	374 (37.8)
70+	664	98 (6.2)	158 (11.2)	194 (13.1)	214 (13.4)
Ethnicity					
White	2016	490 (71.8)	415 (65.7)	486 (71.0)	625 (80.7)
Black	664	166 (9.6)	181 (10.7)	180 (9.6)	137 (6.6)
Mexican-American	623	163 (8.9)	207 (11.1)	173 (8.4)	80 (3.0)
Others	736	156 (9.7)	198 (12.5)	196 (11.0)	186 (9.7)
PIR					
≤1.3	1160	360 (29.0)	299 (21.6)	269 (18.2)	232 (15.2)
>1.3	2543	545 (71.0)	607 (78.4)	673 (81.8)	718 (84.8)
Alcohol consumption ^1^					
None	1487	377 (33.0)	384 (30.9)	377 (29.2)	349 (29.2)
Moderate	1309	241 (26.4)	309 (34.4)	367 (43.8)	392 (40.6)
High	1243	357 (40.6)	308 (34.7)	291 (27.0)	287 (30.2)
Smoking ^2^					
Never	2182	459 (51.1)	555 (57.1)	601 (57.7)	567 (59.4)
Former	1014	212 (22.2)	238 (24.5)	267 (27.6)	297 (26.4)
Current (<15 cigarettes/day)	444	155 (13.9)	109 (11.0)	96 (8.7)	84 (7.5)
Current (≥15 cigarettes/day)	292	115 (12.8)	63 (7.3)	50 (6.0)	64 (6.7)
Physical activity ^3^					
Inactive	1570	451 (42.6)	370 (32.2)	362 (32.4)	387 (34.4)
<500 MET min/week	506	137 (14.9)	129 (14.0)	122 (11.0)	118 (10.8)
≥500 MET min/week	1961	387 (42.5)	502 (53.8)	550 (56.6)	522 (54.8)
BMI (kg/m^2^)					
<25	1295	286 (30.3)	319 (33.3)	358 (37.3)	332 (34.5)
25–30	1371	325 (33.8)	343 (35.7)	352 (33.5)	351 (33.4)
≥30	1373	364 (35.9)	339 (31.0)	325 (29.2)	345 (32.1)
Antioxidant supplement use ^4^					
Yes	1403	74 (10.0)	302 (35.0)	484 (49.2)	543 (51.8)
No	2636	901 (90.0)	699 (65.0)	551 (50.8)	485 (48.2)
Use of blood pressure medication ^5^					
Yes	1313	261 (22.8)	303 (26.7)	374 (32.6)	375 (32.9)
No	2726	714 (77.2)	698 (73.3)	661 (67.4)	653 (67.1)
Use of diabetes medication ^6^					
Yes	257	64 (5.1)	59 (4.8)	54 (2.8)	80 (5.3)
No	3782	911 (94.9)	942 (95.2)	981 (97.2)	948 (94.7)
Saturated fatty acid intake, (g/day)	4039	28.5	26.9	26.4	24.8
Fiber intake (g/day)	4039	14.2	17.8	19.8	18.8

^1^ Alcohol consumption: defined based on the number of drinks of any type of alcoholic beverage per day, with no consumption of drinks as none, no more than 2 drinks/day for men and no more than 1 drink/day for women as moderate, and more than 2 drinks for men and more than 1 drink for women as high intake; ^2^ smoking: former means to have smoked at least 100 cigarettes in entire life, but do not smoke cigarettes now. Current means to have smoked at least 100 cigarettes in entire life and now smoke cigarettes; ^3^ physical activity: inactive means does not do any walking/bicycling, moderate/vigorous recreational activities for at least 10 min continuously in a typical week. MET, metabolic equivalence of tasks; ^4^ yes defined as those who have more than zero for total antioxidant capacity (TAC) from supplements, no as those who have zero for TAC from supplements; ^5^ use of blood pressure medication: yes means to take prescribed medicine for high blood pressure or cholesterol; ^6^ use of diabetes medication: yes means to take insulin or diabetic pills to lower blood sugar. BMI, body mass index; NHANES, National Health and Nutrition Examination Survey; PIR, poverty-income-ratio; VCE, vitamin C equivalent.

The study participants were divided into antioxidant supplement users and non-users and the contribution of individual antioxidants from diet (antioxidant non-users) or diet/supplements (antioxidant supplement users) to TAC was compared (Table 2). Thirty-five percent of the participants reported using antioxidant supplements. The TAC of antioxidant supplement users was much greater than the TAC of antioxidant supplement non-users. Among supplement users, vitamin C from supplements contributed 28.0% of total TAC, and vitamin E and flavonoids from supplements contributed 2.1% and 1.2% of total TAC, respectively. In addition to the significant contribution of antioxidant supplements to total TAC among the supplement users, TAC from diet for supplement users was greater than that of non-users, and the addition of TAC from supplements to TAC from the diet resulted in a considerable difference in TAC between antioxidant supplement users and non-users. Regardless of antioxidant supplement use, flavonoids were the greatest contributor to TAC, followed by vitamin C and PA, in that order.

**Table 2:** Total antioxidant capacity (TAC) from individual antioxidants by antioxidant supplement users and non-users among U.S. adults in the NHANES 2007–2012 (*n* = 4039) ^1^.

Individual Antioxidants	Total Antioxidant Capacity (mg VCE/day) (Range, Median)			
	Antioxidant Supplement Non-Users (*n* = 2636)	Antioxidant Supplement Users (*n* = 1403)		
	TAC	TAC from Diet	TAC from Supplements	TAC
	(0.5–8432.5, 217.6)	(1.0–9296.5, 268.4)	(0.002–4133.0, 99.9)	(10.4–9362.9, 499.7)
	Mean (95% CI)	Mean (95% CI)	Mean (95% CI)	Mean (95% CI)
Vitamin C	80.1 (2.4)	96.1 (4.0)	241.7 (15.3)	337.8 (16.6)
Vitamin E	2.3 (0.0)	2.7 (0.1)	18.3 (1.1)	21.0 (1.1)
Carotenoids	4.3 (0.1)	4.9 (0.2)	0.3 (0.0)	5.2 (0.3)
Flavonoids	399.8 (28.8)	420.6 (28.2)	10.1 (2.4)	430.7 (28.5)
Proanthocyanidins	63.1 (3.9)	68.9 (4.0)	-	68.9 (4.0)

^1^ Antioxidant supplement users are defined as those who have more than zero for TAC from supplements, and non-users are defined as those who have zero for TAC from supplements. VCE, vitamin C equivalents.

Table 3 lists the major sources of TAC among antioxidant supplement users and non-users. Antioxidant supplements are listed as the second highest TAC source for antioxidant supplement users, providing 31% of daily TAC. The top food source was tea, followed by vegetable mixture, orange juice, wine, and berries in both groups. While the TAC of antioxidant supplement users from vegetable mixture, berries, wine, and dark-green vegetables was higher than the TAC of non-users, the non-users had more TAC from fruit drinks, beer, and potatoes than the supplement users.

The association of TAC with consumption of antioxidant-rich food groups was examined (Table 4). After adjusting for total energy intake, greater TAC was significantly associated with higher consumption of total fruit and fruit products, citrus fruit and juices, berries, apples, total vegetables and vegetable products, vegetable mixture, tomatoes, dark-green vegetables, total beverages except water and citrus fruit juice, tea, and wine. Fruit drinks and coffee were not significantly associated with TAC.

Subjects in the lowest quartile of TAC had greater waist circumference and subjects in the lowest quartile of TAC had greater BMI, fasting glucose, insulin, HOMA-IR, and lower HDL-C than those in the higher quartile of TAC (Table 5). Subjects with higher TAC had a lower TG/HDL-C ratio and TG level than those with lower TAC.

Table 6 shows the association of CVD risk factors with TAC from diet, TAC from supplements and TAC from both diet and supplements. For TAC from diet, greater TAC was associated with reduced TG, TG/HDL-C ratio, CRP, and increased HDL-C after adjusting for all variables. The percent changes in TG and TG/HDL-C ratio for a 100% increase in TAC were −1.55% (95% CI = −2.60 to −0.50) and −2.14% (95% CI = −3.43 to −0.83), respectively. The percent changes in CRP and HDL-C for a 100% increase in TAC were −0.68% (95% CI = −1.15 to −0.20) and 0.60% (95% CI = 0.05 to 1.15), respectively. For TAC from supplements, greater TAC was associated with reduced BMI (−0.63% change; 95% CI = −0.86 to −0.39), TC/HDL-C ratio (−0.39% change; 95% CI = −0.71 to −0.08), insulin (−0.98% change; 95% CI = −1.58 to −0.37), and HOMA-IR (−1.09% change; 95% CI = −1.72 to −0.45) after adjusting for all variables. For TAC from both diet and supplements, greater TAC was significantly associated with reduced TG (−1.39% change; 95% CI = −2.56 to −0.21), TG/HDL-C ratio (−2.03% change; 95% CI = −3.45 to −0.60), CRP (−0.83% change; 95% CI = −1.29 to −0.38), insulin (−1.37% change; 95% CI = −2.64 to −0.09), HOMA-IR (−1.57% change; 95% CI = −3.02 to −0.09), and increased HDL-C (0.65% change; 95% CI = 0.07 to 1.23). There was no significant association of TAC with waist circumference, BMI, blood pressure, LDL-C, TC, and fasting glucose.

**Table 3:** Top sources contributing to total antioxidant capacity (TAC) of antioxidant supplement users and non-users among U.S. adults in the NHANES 2007–2012 (*n* = 4039).

Rank	Antioxidant Supplement Users ^1^				Antioxidant Supplement Non-Users ^1^			
	Food Group	TAC (mg VCE/Day)	%	Cum%	Food Group	TAC (mg VCE/Day)	%	Cum%
1	Tea	318.4	36.9%	36.9%	Tea	328.3	60.8%	60.8%
2	Antioxidant supplements	270.4	31.3%	68.2%	Vegetable mixture ^3^	32.8	6.5%	67.4%
3	Vegetable mixture ^2^	52.6	6.1%	74.3%	Orange juice	20.6	4.1%	71.5%
4	Orange juice	25.0	2.9%	77.2%	Wine	11.8	2.3%	73.8%
5	Wine	21.8	2.5%	79.7%	Fruit drink	11.2	2.2%	76.0%
6	Berries	20.7	2.4%	82.1%	Berries	11.1	2.2%	78.3%
7	Apple	9.0	1.0%	83.1%	Grain mixtures, frozen plate meals, soups	8.2	1.6%	79.9%
8	Grain mixtures, frozen plate meals, soups	7.8	0.9%	84.0%	Apple	7.7	1.5%	81.4%
9	Orange	7.6	0.9%	84.9%	Orange	6.7	1.3%	82.8%
10	Dark-green vegetables	7.4	0.9%	85.8%	Tomato	6.3	1.2%	84.0%
11	Fruit drink	7.1	0.8%	86.6%	Beer	5.6	1.1%	85.1%
12	Tomato	6.9	0.8%	87.4%	Potato	5.4	1.1%	86.2%
13	Banana	6.7	0.8%	88.2%	Banana	4.9	1.0%	87.2%
14	Legumes	4.4	0.5%	88.7%	Legumes	4.5	0.9%	88.1%
15	Potato	4.3	0.5%	89.2%	Dark-green vegetables	4.2	0.8%	88.9%

^1^ Antioxidant supplement users are defined as those who have more than zero for TAC from supplements, and non-users are defined as those who have zero for TAC from supplements. Antioxidant supplements include vitamin C, vitamin E, carotenoids, and flavonoids; ^2^ vegetable mixture defined as raw and cooked vegetables and their mixtures other than white potatoes, dark green and deep yellow vegetables, tomatoes, lettuce, green beans, corn, peas, and lima beans. VCE, vitamin C equivalents.

**Table 4:** Major food intakes by energy-adjusted quartiles of total antioxidant capacity among U.S. adults in the NHANES 2007–2012 (g/day) (*n* = 4039).

Intake (g/day)	Total Antioxidant Capacity (mg VCE/day) (Range, Median)				*p*-Value for Linear Trend ^1^
	Q1 (*n* = 975)	Q2 (*n* = 1001)	Q3 (*n* = 1035)	Q4 (*n* = 1028)	
	(0.5–216.6, 74.1)	(65.6–466.8, 188.9)	(142.3–1199.8, 428.9)	(379.7–9362.9, 1219.4)	
	Mean (SEM)	Mean (SEM)	Mean (SEM)	Mean (SEM)	
Fruit & fruit products	51.2 (3.4)	166.2 (7.0)	253.0 (9.5)	201.1 (10.0)	<0.0001
Citrus fruit and juices	5.0 (0.8)	40.0 (2.9)	97.0 (7.4)	65.3 (5.8)	<0.0001
Berries	1.0 (0.3)	4.7 (0.6)	11.6 (1.6)	13.6 (1.7)	<0.0001
Apple	7.7 (1.3)	29.1 (2.5)	28.4 (2.3)	25.0 (2.7)	<0.0001
Vegetables & vegetable products	122.0 (3.6)	158.8 (7.9)	183.6 (6.8)	196.8 (18.0)	<0.0001
Vegetable mixture ^2^	31.0 (2.4)	57.8 (3.2)	72.0 (5.0)	86.0 (16.4)	<0.0001
Tomatoes	24.6 (1.7)	33.3 (3.5)	38.2 (3.1)	38.2 (2.8)	0.0025
Dark-green vegetables	4.6 (1.1)	10.2 (1.1)	14.8 (1.4)	14.9 (1.2)	<0.0001
Tea, coffee & other beverages ^3^	1138.7 (41.9)	957.3 (35.8)	943.8 (30.4)	1222.3 (30.1)	0.0017
Tea	23.0 (8.0)	29.5 (6.2)	110.8 (8.1)	526.2 (23.9)	<0.0001
Wine	4.9 (1.5)	11.3 (2.0)	31.8 (4.9)	34.7 (5.4)	<0.0001
Coffee	326.4 (21.5)	303.3 (18.9)	313.8 (15.9)	276.4 (12.0)	0.2556
Fruit drinks	80.3 (9.9)	104.9 (10.5)	105.2 (11.7)	56.5 (6.1)	0.2599

VCE, vitamin C equivalents; ^1^ adjusted for total energy intake; ^2^ vegetable mixture is defined as raw and cooked vegetables and their mixtures other than white potatoes, dark green and deep yellow vegetables, tomatoes, lettuce, green beans, corn, peas, and lima beans; ^3^ total beverages except water and citrus fruit juice.

**Table 5:** Cardiovascular disease risk factors according to energy-adjusted quartiles of total antioxidant capacity among U.S. adults in the NHANES 2007–2012 (*n* = 4039).

Risk Factors	Total Antioxidant Capacity (mg VCE/day) (Range, Median)				*p*-Value for Linear Trend ^1^
	Q1 (*n* = 975)	Q2 (*n* = 1001)	Q3 (*n* = 1035)	Q4 (*n* = 1028)	
	(0.5–216.6, 74.1)	(65.6–466.8, 188.9)	(142.3–1199.8, 428.9)	(379.7–9362.9, 1219.4)	
	Mean (SEM)	Mean (SEM)	Mean (SEM)	Mean (SEM)	
Waist circumference (cm)	99.1 (0.8)	96.8 (0.8)	97.4 (0.8)	97.5 (0.8)	0.4363
BMI (kg/m^2^)	29.0 (0.3)	28.0 (0.3)	28.0 (0.3)	28.1 (0.3)	0.0544
Blood pressure (mm Hg)					
Systolic	118.4 (0.7)	120.0 (0.8)	120.2 (0.8)	119.9 (0.5)	0.4755
Diastolic	69.3 (0.6)	69.1 (0.7)	69.3 (0.5)	68.7 (0.5)	0.9460
HDL-C (mg/dL)	51.1 (0.6)	53.3 (0.6)	54.8 (0.6)	55.5 (0.8)	0.0289
LDL-C (mg/dL)	116.6 (1.5)	117.5 (1.5)	114.2 (1.8)	118.8 (1.5)	0.7873
TC (mg/dL)	194.1 (1.9)	197.9 (1.7)	193.6 (2.1)	198.5 (1.8)	0.8711
Triglycerides (mg/dL)	134.1 (3.5)	137.7 (4.7)	126.5 (4.2)	123.0 (3.8)	0.0045
TG/HDL-C ratio	3.1 (0.1)	3.1 (0.2)	2.7 (0.1)	2.7 (0.1)	0.0019
TC/HDL-C ratio	4.0 (0.1)	4.0 (0.1)	3.8 (0.1)	3.8 (0.1)	0.1055
Fasting glucose (mg/dL)	105.4 (0.9)	104.2 (1.2)	102.8 (0.8)	103.4 (0.8)	0.1158
Insulin (pmol/L)	82.4 (2.6)	75.4 (2.2)	73.1 (2.5)	74.1 (3.6)	0.0781
HOMA-IR	3.7 (0.1)	3.5 (0.1)	3.2 (0.1)	3.3 (0.2)	0.0617
	Q1 (*n* = 681)	Q2 (*n* = 694)	Q3 (*n* = 710)	Q4 (*n* = 709)	
	(2.3–216.6, 75.3)	(65.6–466.8, 186.8)	(162.6–1199.8, 416.8)	(379.7–9362.9, 1237.3)	
CRP ^3^ (mg/L)	1.9 (0.1)	1.4 (0.1)	1.4 (0.1)	1.5 (0.1)	0.0092

^1^ Test for linearity of the trend was done after adjusting for total energy intake, age, gender, ethnicity, physical activity, PIR, smoking, alcohol consumption, blood pressure medication, diabetes medication, and BMI. Model of BMI was adjusted for all variables except BMI. For TG, HDL-C, LDL-C, TC, TG/HDL-C, and TC/HDL-C ratio, the model was additionally adjusted for saturated fatty acid and fiber intakes; ^3^ NHANES 2007–2010 (*n* = 2794); values are geometric means and SEM. HOMA-IR, homeostasis model assessment of insulin resistance; VCE, vitamin C equivalents.

**Table 6:** Association between cardiovascular disease risk factors and total antioxidant capacity (TAC) among U.S. adults in the NHANES 2007–2012 (*n* = 4039) ^1^.

Risk Factors	% Change Predicted in CVD Risk Factor with a 100% Increase Total Antioxidant Capacity (95% CI)					
	TAC_diet_	*p*-Value	TAC_supplements_	*p*-Value	TAC_diet + supplement_	
	%Δ (95% CI)		%Δ (95% CI)		%Δ (95% CI)	*p*-Value
Waist circumference ^2^	−0.12 (−0.26, 0.03)	0.1150	−0.02 (−0.08, 0.04)	0.5452	−0.12 (−0.25, 0.01)	0.0664
BMI ^2^	−0.05 (−0.64, 0.54)	0.8623	−0.63 (−0.86, −0.39)	<0.0001	−0.42 (−1.03, 0.20)	0.1883
Blood Pressure ^2^						
Systolic	−0.06 (−0.37, 0.25)	0.7071	−0.07 (−0.20, 0.06)	0.2674	−0.04 (−0.33, 0.26)	0.8106
Diastolic	0.13 (−0.39, 0.66)	0.6149	−0.11 (−0.32, 0.10)	0.2988	0.13 (−0.39, 0.65)	0.6315
HDL-C ^3^	0.60 (0.05, 1.15)	0.0368	0.31 (−0.03, 0.65)	0.0835	0.65 (0.07, 1.23)	0.0315
LDL-C ^3^	0.09 (−0.66, 0.84)	0.8142	−0.29 (−0.59, 0.00)	0.0595	0.07 (−0.67, 0.81)	0.8594
TC ^3^	0.02 (−0.55, 0.60)	0.9404	−0.09 (−0.29, 0.12)	0.4145	0.05 (−0.51, 0.61)	0.8675
TG ^3^	−1.55 (−2.60, −0.50)	0.0059	−0.17 (−0.66, 0.33)	0.5144	−1.39 (−2.56, −0.21)	0.0251
TG/HDL-C ratio ^3^	−2.14 (−3.43, −0.83)	0.0025	−0.47 (−1.19, 0.25)	0.2025	−2.03 (−3.45, −0.60)	0.0079
TC/HDL-C ratio ^3^	−0.57 (−1.22, 0.08)	0.0929	−0.39 (−0.71, −0.08)	0.0186	−0.60 (−1.30, 0.10)	0.0982
Fasting glucose ^2^	−0.12 (−0.41, 0.18)	0.4403	−0.12 (−0.26, 0.02)	0.1095	−0.20 (−0.51, 0.12)	0.2297
Insulin ^2^	−0.57 (−1.96, 0.83)	0.4262	−0.98 (−1.58, −0.37)	0.0028	−1.37 (−2.64, −0.09)	0.0415
HOMA-IR ^2^	−0.71 (−2.27, 0.88)	0.3851	−1.09 (−1.72, −0.45)	0.0017	−1.57 (−3.02, −0.09)	0.0428
CRP ^2^	−0.68 (−1.15, −0.20)	0.0090	−0.18 (−0.50, 0.15)	0.2892	−0.83 (−1.29, −0.38)	0.0012

^1^ Multivariate linear regression analysis of cardiovascular disease risk factors. Values are changes in percentages (%) of cardiovascular disease risk factors with a 100% increase of TAC; ^2^ model 1: Adjusted for total energy intake, age, gender, ethnicity, physical activity, PIR, smoking, alcohol consumption, blood pressure medication, diabetes medication, and BMI. BMI was adjusted for all variables except BMI; ^3^ model 2: Model 1 + adjusted for saturated fatty acid and fiber intake. BMI, body mass index; CRP, C-reactive protein; HDL, high-density lipoprotein; HOMA-IR, homeostasis model assessment of insulin resistance; LDL, low-density lipoprotein; TC, total cholesterol; TG, triglycerides.

## 4. Discussion

This study found that TAC is associated with reduced blood CVD risk factors. Both greater TAC from diet and TAC from diet and supplements were associated with reduced blood TG, TG/HDL-C ratio, CRP, and increased HDL-C after adjusting for all covariates. Since levels of cholesterol and TG may be affected by saturated fat and fiber intake [40,41], cholesterols and TG were additionally adjusted for these dietary variables in regression model 2. These results are consistent with a Spanish study showing an inverse association between TAC and TG, oxidized LDL-C, hyperglycemia, and positive association with HDL-C in 266 healthy young adults [17]. Our results are also in accordance with a study showing that the consumption of cocoa rich in flavonoids was effective in lowering TG by blocking the COX-2 activation and reducing synthesis of inflammatory prostanoids [42]. The association of TAC with HDL-C is also supported by the study that reported positive effects of flavonoid-rich cocoa powder and orange juice on HDL-C in human intervention trials [43,44]. Since flavonoids are a major contributor to TAC, these findings are also supported by the previous report that higher total flavonoid intake is associated with lower serum TG and TG/HDL-C ratio [45]. The inverse association of TAC with CRP in the present study is consistent with our previous study reporting an inverse association of TAC with serum CRP and plasma homocysteine using NHANES 2001–2002 [20].

In the present study, antioxidant supplements were identified as a second top source contributing to TAC. Although several randomized controlled trials have failed to support the beneficial effects of antioxidant supplements on CVD risk [46,47], many observational studies have identified an inverse association of antioxidant supplement use with CVD risk [48,49]. These protective effects of antioxidants have also been supported by studies showing that antioxidants play a key role in inhibiting oxidative damage and lipid oxidation reactions that lead to atherosclerosis and CVD [50]. Considering the great extent to which antioxidant supplements contribute to TAC and CVD risk factors and the high prevalence of dietary supplement use in the United States [18], TAC from supplements should be included in the study of the association of TAC with CVD risk. However, most previous studies on antioxidant and CVD risk have estimated antioxidants from the diet only [11,12,13,14,17] or from one or a mixture of several antioxidant supplements [51].

In addition, most previous studies estimated TAC by utilizing food TAC databases, in which the TAC value of a limited number of food items were determined by several assays, such as total ferric reducing antioxidant power (FRAP) or oxygen radical absorbance capacity (ORAC) [52,53,54,55,56]. To overcome the limitations of the previous approaches, our research group first developed and validated an algorithm to estimate the TAC of the U.S. diet [29]. The vitamin C equivalent antioxidant capacity (VCEAC) of individual antioxidant nutrients and 50 popular antioxidant-rich food items in the U.S. diet were determined by ABTS assay and 1,1-diphenyl-2-picrylhydrazyl (DPPH) assay. According to the algorithm, the theoretical TAC for each food item was calculated as the sum of the product of antioxidant content and antioxidant capacities of individual antioxidants. Theoretical TAC was positively correlated with experimental TAC values measured by the ABTS assay (*r* = 0.833, *p* < 0.001), the DPPH assay (*r* = 0.696, *p* < 0.001), and TAC from the USDA ORAC database (*r* = 0.484, *p* < 0.001). This investigative protocol has been further validated by comparing individual antioxidant intakes with their concentrations in plasma [57] and urine collections [58]. In our recent human observational studies, TAC was validated for its relevance, reliability, and predictability for *in vivo* antioxidant status under different physiological conditions [59,60,61]. This theoretical TAC enables us to estimate TAC from supplements as well as TAC from diet, and to calculate the contribution of individual antioxidants to TAC.

In this study, the results obtained with TAC from both diet and supplements were different from the results obtained with TAC from diet only. When we considered TAC from both diet and supplements, we found an additional inverse association of TAC with insulin and HOMA-IR as well as the association of TAC with improved TG, TG/HDL-C ratio, CRP, and HDL-C. As vitamin C is a major contributor to TAC from supplements, this finding is supported by the report that vitamin C supplementation had beneficial effects on lowering fasting insulin levels by improving endothelium-dependent vasodilation in patients with non-insulin-dependent diabetes mellitus [62,63].

Our results show that greater TAC is strongly associated with higher consumption of fruits and fruit products, vegetables and vegetable products, beverages, tea, and wine, which have been known to be inversely associated with CVD risk [64,65]. We found strong positive associations of TAC with high consumption of citrus fruits and fruit juices [66], berries [67], apple [68], vegetable mixture, and dark-green vegetables [69], which have been reported to be negatively associated with TG, TC, and TC/HDL-C ratio in previous studies. Greater TAC was associated with higher consumption of tea and wine, which was inversely associated with insulin level, insulin resistance, LDL, TG, inflammation, and oxidative stress [70]. Our findings of the association of greater TAC with improved CVD risk factors could be explained through positive associations of TAC with fruit, vegetable, tea, and wine consumption.

This study has several strengths. First, we used a large sample of the representative U.S. adult population. Second, we used an improved flavonoid database, which provides more complete data and better estimates of flavonoid intake. Third, we estimated TAC from supplements as well as TAC from diet. However, the present study also has limitations. First, no causal inference can be drawn because this study was based on cross-sectional data. Second, we did not consider the bioavailability and *in vivo* activity of dietary antioxidant compounds. Third, the estimation of TAC was based on two 24-h dietary recalls, which may not provide accurate estimates of the usual intake of participants. Fourth, there may still be residual confounding factors, although attempts were made in our two models to control for all relevant factors for which data were available.

## 5. Conclusions

In conclusion, greater TAC was associated with improved TG, TG/HDL-C ratio, HDL-C, insulin, HOMA-IR, and CRP. Our findings support the hypothesis that an antioxidant-rich diet and intake of supplements are beneficial in reducing CVD risk.

## Abbreviations

The following abbreviations are used in this manuscript:

BMI: body mass index
CRP: C-reactive protein
CVD: cardiovascular disease
EPIC: European Prospective Investigation into Cancer and Nutrition
FRAP: ferric reducing antioxidant power
HDL: high-density lipoprotein
HDL-C: high-density lipoprotein cholesterol
HOMA-IR: homeostasis model assessment of insulin resistance
LDL: low-density lipoprotein
LDL-C: low-density lipoprotein cholesterol
METs: metabolic equivalence of tasks
ORAC: oxygen radical absorbance capacity
PA: proanthocyanidin
PIR: poverty-income-ratio
ROS: reactive oxygen species
TAC: total antioxidant capacity
TC: total cholesterol
TEAC: Trolox equivalent antioxidant capacity
TG: triglycerides
VCE: vitamin C equivalents
VCEAC: vitamin C equivalent antioxidant capacity

## References

[1] Steinberg D. (1997). Low density lipoprotein oxidation and its pathobiological significance. J. Biol. Chem..

[2] Bhupathiraju S.N., Wedick N.M., Pan A., Manson J.E., Rexrode K.M., Willett W.C., Rimm E.B., Hu F.B. (2013). Quantity and variety in fruit and vegetable intake and risk of coronary heart disease. Am. J. Clin. Nutr..

[3] Liu R.H. (2003). Health benefits of fruit and vegetables are from additive and synergistic combinations of phytochemicals. Am. J. Clin. Nutr..

[4] McCullough M.L., Peterson J.J., Patel R., Jacques P.F., Shah R., Dwyer J.T. (2012). Flavonoid intake and cardiovascular disease mortality in a prospective cohort of us adults. Am. J. Clin. Nutr..

[5] Osganian S.K., Stampfer M.J., Rimm E., Spiegelman D., Manson J.E., Willett W.C. (2003). Dietary carotenoids and risk of coronary artery disease in women. Am. J. Clin. Nutr..

[6] Cook N.R., Albert C.M., Gaziano J.M., Zaharris E., MacFadyen J., Danielson E., Buring J.E., Manson J.E. (2007). A randomized factorial trial of vitamins C and E and beta carotene in the secondary prevention of cardiovascular events in women: Results from the women’s antioxidant cardiovascular study. Arch. Intern. Med..

[7] Hennekens C.H., Buring J.E., Manson J.E., Stampfer M., Rosner B., Cook N.R., Belanger C., LaMotte F., Gaziano J.M., Ridker P.M. (1996). Lack of effect of long-term supplementation with beta carotene on the incidence of malignant neoplasms and cardiovascular disease. N. Engl. J. Med..

[8] Harasym J., Oledzki R. (2014). Effect of fruit and vegetable antioxidants on total antioxidant capacity of blood plasma. Nutrition.

[9] Barron B.A. (1977). The effects of misclassification on the estimation of relative risk. Biometrics.

[10] Serafini M., del Rio D. (2004). Understanding the association between dietary antioxidants, redox status and disease: Is the total antioxidant capacity the right tool?. Redox Rep..

[11] Puchau B., Zulet M.A., de Echavarri A.G., Hermsdorff H.H., Martinez J.A. (2009). Dietary total antioxidant capacity: A novel indicator of diet quality in healthy young adults. J. Am. Coll. Nutr..

[12] Rautiainen S., Larsson S., Virtamo J., Wolk A. (2012). Total antioxidant capacity of diet and risk of stroke: A population-based prospective cohort of women. Stroke.

[13] Del Rio D., Agnoli C., Pellegrini N., Krogh V., Brighenti F., Mazzeo T., Masala G., Bendinelli B., Berrino F., Sieri S. (2011). Total antioxidant capacity of the diet is associated with lower risk of ischemic stroke in a large Italian cohort. J. Nutr..

[14] Rautiainen S., Levitan E.B., Mittleman M.A., Wolk A. (2013). Total antioxidant capacity of diet and risk of heart failure: A population-based prospective cohort of women. Am. J. Med..

[15] Franzini L., Ardigo D., Valtuena S., Pellegrini N., del Rio D., Bianchi M.A., Scazzina F., Piatti P.M., Brighenti F., Zavaroni I. (2012). Food selection based on high total antioxidant capacity improves endothelial function in a low cardiovascular risk population. Nutr. Metab. Cardiovasc. Dis..

[16] Valtuena S., Pellegrini N., Franzini L., Bianchi M.A., Ardigo D., del Rio D., Piatti P., Scazzina F., Zavaroni I., Brighenti F. (2008). Food selection based on total antioxidant capacity can modify antioxidant intake, systemic inflammation, and liver function without altering markers of oxidative stress. Am. J. Clin. Nutr..

[17] Hermsdorff H.H., Puchau B., Volp A.C., Barbosa K.B., Bressan J., Zulet M.A., Martinez J.A. (2011). Dietary total antioxidant capacity is inversely related to central adiposity as well as to metabolic and oxidative stress markers in healthy young adults. Nutr. Metab..

[18] Bailey R.L., Gahche J.J., Lentino C.V., Dwyer J.T., Engel J.S., Thomas P.R., Betz J.M., Sempos C.T., Picciano M.F. (2011). Dietary supplement use in the United States, 2003–2006. J. Nutr..

[19] Yang M., Chung S.J., Chung C.E., Kim D.O., Song W.O., Koo S.I., Chun O.K. (2011). Estimation of total antioxidant capacity from diet and supplements in USA adults. Br. J. Nutr..

[20] Yang M., Chung S.J., Floegel A., Song W.O., Koo S.I., Chun O.K. (2013). Dietary antioxidant capacity is associated with improved serum antioxidant status and decreased serum C-reactive protein and plasma homocysteine concentrations. Eur. J. Nutr..

[21] Wang Y., Yang M., Lee S.G., Davis C.G., Koo S.I., Fernandez M.L., Volek J.S., Chun O.K. (2014). Diets high in total antioxidant capacity improve risk biomarkers of cardiovascular disease: A 9-month observational study among overweight/obese postmenopausal women. Eur. J. Nutr..

[22] National Center for Health Statistics (2010). National Health and Nutrition Examination Survey Questionnaire, 2007–2008 Data Files.

[23] National Center for Health Statistics (2012). National Health and Nutrition Examination Survey Questionnaire, 2009–2010 Data Files.

[24] National Center for Health Statistics (2014). National Health and Nutrition Examination Survey Questionnaire, 2011–2012 Data Files.

[25] Agricultural Research Service (2013). USDA Database for the Flavonoid Content of Selected Foods, Release 3.1.

[26] Agricultural Research Service (2008). USDA Database for the Isoflavone Content of Selected Foods, Release 2.0.

[27] Agricultural Research Service (2004). USDA Database for the Proanthocyanidin Content of Selected Foods.

[28] Chun O.K., Chung S.J., Song W.O. (2007). Estimated dietary flavonoid intake and major food sources of USA adults. J. Nutr..

[29] Floegel A., Kim D.O., Chung S.J., Song W.O., Fernandez M.L., Bruno R.S., Koo S.I., Chun O.K. (2010). Development and validation of an algorithm to establish a total antioxidant capacity database of the USA diet. Int. J. Food. Sci. Nutr..

[30] Sinha S.K., Ghaskadbi S.S. (2013). Thearubigins rich black tea fraction reveals strong antioxidant activity. Int. J. Green Pharm..

[31] Ahuja J.K.C., Montville J.B., Omolewa-Tomobi G., Heendeniya K.Y., Martin C.L., Steinfeldt L.C., Anand J., Adler M.E., LaComb R.P., Moshfegh A.J. (2012). USDA Food and Nutrient Database for Dietary Studies, 5.0.

[32] National Center for Health Statistics (2009). 2009–2010 National Health and Nutrition Examination Survey, Anthropometry Procedures Manual.

[33] National Center for Health Statistics (2009). 2009–2010 National Health and Nutrition Examination Survey, Laboratory Procedures Manual.

[34] Matthews D.R., Hosker J.P., Rudenski A.S., Naylor B.A., Treacher D.F., Turner R.C. (1985). Homeostasis model assessment: Insulin resistance and beta-cell function from fasting plasma glucose and insulin concentrations in man. Diabetologia.

[35] Lemieux I., Lamarche B., Couillard C., Pascot A., Cantin B., Bergeron J., Dagenais G.R., Despres J.P. (2001). Total cholesterol/HDL cholesterol ratio *vs.* LDL cholesterol/HDL cholesterol ratio as indices of ischemic heart disease risk in men: The Quebec Cardiovascular Study. Arch. Intern. Med..

[36] Da Luz P.L., Favarato D., Faria-Neto J.R., Lemos P., Chagas A.C. (2008). High ratio of triglycerides to HDL-cholesterol predicts extensive coronary disease. Clinics.

[37] Willett W.C., Howe G.R., Kushi L.H. (1997). Adjustment for total energy intake in epidemiologic studies. Am. J. Clin. Nutr..

[38] Krauss R.M., Eckel R.H., Howard B., Appel L.J., Daniels S.R., Deckelbaum R.J., Erdman J.W., Kris-Etherton P., Goldberg I.J., Kotchen T.A. (2000). AHA dietary guidelines: Revision 2000: A statement for healthcare professionals from the nutrition committee of the American Heart Association. Circulation.

[39] Ainsworth B.E., Haskell W.L., Herrmann S.D., Meckes N., Bassett D.R., Tudor-Locke C., Greer J.L., Vezina J., Whitt-Glover M.C., Leon A.S. (2011). 2011 compendium of physical activities: A second update of codes and met values. Med. Sci. Sports Exerc..

[40] Jenkins D.J., Wolever T.M., Rao A.V., Hegele R.A., Mitchell S.J., Ransom T.P., Boctor D.L., Spadafora P.J., Jenkins A.L., Mehling C. (1993). Effect on blood lipids of very high intakes of fiber in diets low in saturated fat and cholesterol. N. Engl. J. Med..

[41] Anderson J.W. (2000). Dietary fiber prevents carbohydrate-induced hypertriglyceridemia. Curr. Atheroscler. Rep..

[42] Parsaeyan N., Mozaffari-Khosravi H., Absalan A., Mozayan M.R. (2014). Beneficial effects of cocoa on lipid peroxidation and inflammatory markers in type 2 diabetic patients and investigation of probable interactions of cocoa active ingredients with prostaglandin synthase-2 (PTGS-2/COX-2) using virtual analysis. J. Diabetes Metab. Disord..

[43] Kurowska E.M., Spence J.D., Jordan J., Wetmore S., Freeman D.J., Piche L.A., Serratore P. (2000). HDL-cholesterol-raising effect of orange juice in subjects with hypercholesterolemia. Am. J. Clin. Nutr..

[44] Mursu J., Voutilainen S., Nurmi T., Rissanen T.H., Virtanen J.K., Kaikkonen J., Nyyssonen K., Salonen J.T. (2004). Dark chocolate consumption increases HDL cholesterol concentration and chocolate fatty acids may inhibit lipid peroxidation in healthy humans. Free Radic. Biol. Med..

[45] Li G., Zhu Y., Zhang Y., Lang J., Chen Y., Ling W. (2013). Estimated daily flavonoid and stilbene intake from fruits, vegetables, and nuts and associations with lipid profiles in Chinese adults. J. Acad. Nutr. Diet..

[46] Virtamo J., Rapola J.M., Ripatti S., Heinonen O.P., Taylor P.R., Albanes D., Huttunen J.K. (1998). Effect of vitamin E and beta carotene on the incidence of primary nonfatal myocardial infarction and fatal coronary heart disease. Arch. Intern. Med..

[47] Sesso H.D., Buring J.E., Christen W.G., Kurth T., Belanger C., MacFadyen J., Bubes V., Manson J.E., Glynn R.J., Gaziano J.M. (2008). Vitamins E and C in the prevention of cardiovascular disease in men: The physicians’ health study II randomized controlled trial. JAMA.

[48] Pocobelli G., Peters U., Kristal A.R., White E. (2009). Use of supplements of multivitamins, vitamin C, and vitamin E in relation to mortality. Am. J. Epidemiol..

[49] Buijsse B., Feskens E.J., Kwape L., Kok F.J., Kromhout D. (2008). Both alpha- and beta-carotene, but not tocopherols and vitamin C, are inversely related to 15-year cardiovascular mortality in Dutch elderly men. J. Nutr..

[50] Zhang P.Y., Xu X., Li X.C. (2014). Cardiovascular diseases: Oxidative damage and antioxidant protection. Eur. Rev. Med. Pharmacol. Sci..

[51] Mekary R.A., Wu K., Giovannucci E., Sampson L., Fuchs C., Spiegelman D., Willett W.C., Smith-Warner S.A. (2010). Total antioxidant capacity intake and colorectal cancer risk in the health professionals follow-up study. Cancer Causes Control.

[52] Halvorsen B.L., Carlsen M.H., Phillips K.M., Bohn S.K., Holte K., Jacobs D.R., Blomhoff R. (2006). Content of redox-active compounds (*i.e.*, antioxidants) in foods consumed in the United States. Am. J. Clin. Nutr..

[53] Halvorsen B.L., Holte K., Myhrstad M.C., Barikmo I., Hvattum E., Remberg S.F., Wold A.B., Haffner K., Baugerod H., Andersen L.F. (2002). A systematic screening of total antioxidants in dietary plants. J. Nutr..

[54] Pellegrini N., Serafini M., Colombi B., del Rio D., Salvatore S., Bianchi M., Brighenti F. (2003). Total antioxidant capacity of plant foods, beverages and oils consumed in Italy assessed by three different *in vitro* assays. J. Nutr..

[55] Pellegrini N., Serafini M., Salvatore S., del Rio D., Bianchi M., Brighenti F. (2006). Total antioxidant capacity of spices, dried fruits, nuts, pulses, cereals and sweets consumed in Italy assessed by three different *in vitro* assays. Mol. Nutr. Food Res..

[56] Salvatore S., Pellegrini N., Brenna O.V., del Rio D., Frasca G., Brighenti F., Tumino R. (2005). Antioxidant characterization of some Sicilian edible wild greens. J. Agric. Food Chem..

[57] Wang Y., Chung S.J., McCullough M.L., Song W.O., Fernandez M.L., Koo S.I., Chun O.K. (2014). Dietary carotenoids are associated with cardiovascular disease risk biomarkers mediated by serum carotenoid concentrations. J. Nutr..

[58] Chun O.K., Chung S.J., Song W.O. (2009). Urinary isoflavones and their metabolites validate the dietary isoflavone intakes in USA adults. J. Am. Diet. Assoc..

[59] Wang Y., Yang M., Davis C.G., Koo S.I., Chun O.K. (2011). Dietary total antioxidant capacity reflects dietary and plasma antioxidant status in healthy college students. FASEB J..

[60] Wang Y., Yang M., Lee S.-G., Davis C., Masterjohn C., Kenny A., Bruno R.S., Chun O.K., Diederich M., Noworyta K. (2012). Total antioxidant capacity: A useful tool in assessing antioxidant intake status. Natural Compounds as Inducers of Cell Death.

[61] Wang Y., Yang M., Lee S.G., Davis C.G., Kenny A., Koo S.I., Chun O.K. (2012). Plasma total antioxidant capacity is associated with dietary intake and plasma level of antioxidants in postmenopausal women. J. Nutr. Biochem..

[62] Paolisso G., Balbi V., Volpe C., Varricchio G., Gambardella A., Saccomanno F., Ammendola S., Varricchio M., D’Onofrio F. (1995). Metabolic benefits deriving from chronic vitamin C supplementation in aged non-insulin dependent diabetics. J. Am. Coll. Nutr..

[63] Ting H.H., Timimi F.K., Boles K.S., Creager S.J., Ganz P., Creager M.A. (1996). Vitamin C improves endothelium-dependent vasodilation in patients with non-insulin-dependent diabetes mellitus. J. Clin. Investig..

[64] Hung H.C., Joshipura K.J., Jiang R., Hu F.B., Hunter D., Smith-Warner S.A., Colditz G.A., Rosner B., Spiegelman D., Willett W.C. (2004). Fruit and vegetable intake and risk of major chronic disease. J. Natl. Cancer Inst..

[65] Yuan C., Lee H.J., Shin H.J., Stampfer M.J., Cho E. (2015). Fruit and vegetable consumption and hypertriglyceridemia: Korean national health and nutrition examination surveys (KNHANES) 2007–2009. Eur. J. Clin. Nutr..

[66] O’Neil C.E., Nicklas T.A., Rampersaud G.C., Fulgoni V.L. (2012). 100% orange juice consumption is associated with better diet quality, improved nutrient adequacy, decreased risk for obesity, and improved biomarkers of health in adults: National health and nutrition examination survey, 2003–2006. Nutr. J..

[67] Lee I.T., Chan Y.C., Lin C.W., Lee W.J., Sheu W.H. (2008). Effect of cranberry extracts on lipid profiles in subjects with type 2 diabetes. Diabet. Med..

[68] Vafa M.R., Haghighatjoo E., Shidfar F., Afshari S., Gohari M.R., Ziaee A. (2011). Effects of apple consumption on lipid profile of hyperlipidemic and overweight men. Int. J. Prev. Med..

[69] Takahashi K., Kamada C., Yoshimura H., Okumura R., Iimuro S., Ohashi Y., Araki A., Umegaki H., Sakurai T., Yoshimura Y. (2012). Effects of total and green vegetable intakes on glycated hemoglobin A1c and triglycerides in elderly patients with type 2 diabetes mellitus: The Japanese elderly intervention trial. Geriatr. Gerontol. Int..

[70] Bogdanski P., Suliburska J., Szulinska M., Stepien M., Pupek-Musialik D., Jablecka A. (2012). Green tea extract reduces blood pressure, inflammatory biomarkers, and oxidative stress and improves parameters associated with insulin resistance in obese, hypertensive patients. Nutr. Res..

